# Myocardial function recovery in peripartum cardiomyopathy: the role of global longitudinal strain and myocardial work

**DOI:** 10.3389/fcvm.2026.1860070

**Published:** 2026-08-26

**Authors:** Federica Ilardi, Federica Carusone, Dario D’Alconzo, Rachele Manzo, Alessia Palmentieri, Ciro Battaglia, Dalila Nappa, Giuseppe Giugliano, Raffaella Lombardi, Santo Dellegrottaglie, Viviana Narciso, Roberta Paolillo, Luca Bardi, Francesco S. Loffredo, Daniele Masarone, Enrica Pezzullo, Francesca Lanni, Donato Gerardi, Eugenio Stabile, Angelicarosa Cascone, Nouha Azaza, Giovanni Peretto, Giuseppe Bifulco, Attilio Di Spiezio Sardo, Gabriele Saccone, Alaide Chieffo, Cinzia Perrino, Giovanni Esposito

**Affiliations:** 1 Department of Advanced Biomedical Sciences, Federico II University, Naples, Italy; 2 Federico II University Hospital, Naples, Italy; 3 Advanced Cardiovascular Imaging Unit, Clinica Villa dei Fiori, Acerra (Naples), Italy; 4 University of Campania Luigi Vanvitelli, Naples, Italy; 5 Monaldi Hospital, Naples, Italy; 6 S. G. Moscati Hospital, Avellino, Italy; 7 Azienda Ospedaliera Regionale “San Carlo,” Potenza, Italy; 8 Vita-Salute San Raffaele University, Milan, Italy; 9 IRCCS San Raffaele Scientific Institute, Milan, Italy; 10 Department of Public Health, Federico II University, Naples, Italy

**Keywords:** global longitudinal strain, left ventricular systolic dysfunction, myocardial work, myocardial work efficiency, peripartum cardiomyopathy

## Abstract

**Introduction:**

Peripartum cardiomyopathy (PPCM) occurs during the peripartum period or shortly thereafter and is characterised by left ventricular (LV) systolic dysfunction. Although some women achieve recovery of LV ejection fraction (LVEF), others develop persistent systolic impairment. Data on myocardial mechanics and contractile performance after the acute phase are limited.

**Aim:**

We aimed to evaluate whether global longitudinal strain (GLS) and myocardial work (MW) indices could provide a more comprehensive assessment of myocardial performance and identify subtle abnormalities associated with incomplete myocardial recovery in PPCM.

**Methods:**

We retrospectively analysed 22 women (mean age 42.1 ± 8.9 years) with PPCM, evaluated ≥6 months after the acute phase, enrolled in the Italian Multicentre Observational PPCM Registry (NCT05878041). Myocardial mechanics were assessed using GLS, global work index, global constructive work, global wasted work, and global work efficiency (GWE). Patients were stratified by LVEF into Group A (<50%) and Group B (≥50%) and compared with 21 age-matched healthy women (Group C).

**Results:**

LV recovery was observed in 16 women (72.7%). Compared with controls, Groups A and B displayed larger LV diameters and volumes (*p* < 0.001), impaired tricuspid annular plane systolic excursion (*p* = 0.007), and left atrial strain (*p* < 0.001). Group A exhibited significantly worse GLS and MW parameters (*p* < 0.001 for all), consistent with the impairment of LVEF. Despite LVEF recovery, Group B still showed reduced GLS (18.0 ± 3.1% vs. 21.8 ± 1.7%, *p* < 0.001) and GWE (92.6 ± 6.0 vs. 95.9 ± 2.0%, *p* = 0.023) compared with controls.

**Conclusions:**

GLS and MW indices enable a refined assessment of myocardial performance in PPCM. Persistent abnormalities despite normalised LVEF suggest residual subclinical dysfunction and support their potential role as sensitive markers of myocardial impairment beyond conventional measures of systolic function.

## 1 Introduction

Peripartum cardiomyopathy (PPCM) is a rare but potentially life-threatening condition that occurs mainly during the peripartum period or in the months following delivery, pregnancy termination, or miscarriage. It is characterised by signs and symptoms of heart failure (HF) associated with reduced left ventricular ejection fraction (LVEF <45%), in the absence of any other known causes of HF, such as pre-existing cardiomyopathy, valvular heart disease, or congenital heart disease (1). Although approximately 50% of women experience a recovery of LVEF, PPCM remains associated with significant morbidity and mortality. It has been hypothesised that, in some patients, despite recovery, a subclinical systolic dysfunction might persist, potentially affecting prognosis (2–4). Beyond LVEF, other echocardiographic measurements such as global longitudinal strain (GLS) and myocardial work (MW) indices have demonstrated better performance in the evaluation of LV systolic mechanics across several pathologic conditions. To date, little is known about myocardial performance in women who experience PPCM. This study aimed to evaluate the impact of PPCM on GLS and MW indices in patients with PPCM diagnosis, in the absence or presence of subsequent LVEF improvement, and to assess whether these echocardiographic parameters could represent reliable and sensitive markers of persistent residual LV dysfunction.

## 2 Methods

### 2.1 Study cohort

All patients with a confirmed diagnosis of PPCM enrolled in the Italian multicentre observational registry of PPCM (NCT05878041) who had undergone complete clinical and echocardiographic evaluation at least 6 months after the acute phase were considered eligible for the present analysis. Patients were excluded if advanced deformation analysis could not be reliably performed. Specific exclusion criteria included echocardiographic examinations acquired using ultrasound systems not compatible with myocardial work analysis, lack of image availability for offline postprocessing, and suboptimal image quality or inadequate acoustic windows precluding reliable strain and/or MW assessment. Details of the study design of the Italian multicentre registry have been reported previously (5, 6). The registry included 40 women with a history or new diagnosis of PPCM between May 2023 and December 2025. The study was supported by the Italian National Recovery and Resilience Plan (PNRR), project PNRR-MR1-2022-12376858, funded by the European Union (NextGenerationEU). The local institutional ethics committee approved the study protocol, and all patients provided written informed consent to participate.

### 2.2 Echocardiographic assessment and data collection

Clinical, echocardiographic, and laboratory variables were collected and recorded in a dedicated database. Echocardiographic acquisitions were performed at the participating centres by experienced echocardiographers following a standardised acquisition protocol, using a Vivid ultrasound (E95) System (GE Healthcare, Horten, Norway). Images were stored on a dedicated workstation (EchoPAC, GE Healthcare, Version 206). All speckle-tracking and MW analyses were performed offline by a single experienced operator blinded to clinical data and recovery status, in order to minimise interobserver variability. For each measurement, at least two cardiac cycles were averaged. Conventional echocardiographic measurements were performed in accordance with current guideline recommendations (7, 8). LVEF was calculated using the biplane Simpson method. Patients were categorised according to follow-up LVEF: Group A, LVEF < 50%; Group B, LVEF ≥ 50%. Twenty-one healthy age-matched women without cardiovascular disease, hypertension, diabetes, or structural heart disease were included as controls (Group C). To avoid the influence of physiological postpartum remodelling, women within 12 months after delivery were excluded.

### 2.3 Speckle-tracking-derived global longitudinal strain and myocardial work indices

Strain analysis was based on a speckle-tracking approach and measured by an experienced cardiologist, as previously described (9). LV GLS was performed from the standard apical long-axis, four- and two-chamber views (frame rate 60–90 frames/s). Right ventricle (RV) longitudinal strain was obtained from the apical four-chamber RV-focused view. RV-GLS was calculated as the average of the segments of the RV free wall and interventricular septum; RV free wall longitudinal strain was calculated as the average of free wall segments (10). Peak atrial longitudinal strain (PALS) reflected the left atrial (LA) reservoir phase and was obtained as the average of peak positive strain value during ventricular systole measured in apical four- and two-chamber views, using the R-wave as the reference point (11). Quantification of MW was performed using a commercially available software package (Echopac Version 204, GE Healthcare). As described previously (12, 13), MW was estimated as the area of the pressure–strain loops, which were calculated by integrating LV GLS data with estimated LV pressure from brachial artery blood pressure measured with a cuff manometer. The following MW indices were calculated: global work index (GWI, mmHg %), total work within the area of the pressure–strain loop, calculated during systole; global constructive work (GCW, mmHg %), defined as work performed during myocardial shortening in systole plus lengthening during isovolumetric relaxation; global wasted work (GWW, mmHg %), defined as work performed during myocardial lengthening in systole plus shortening during isovolumetric relaxation; and global work efficiency (GWE, %), the ratio of constructive work to the sum of constructive and wasted work. Reference values for MW indices were derived from previously published data (14).

### 2.4 Statistical analysis

Data are reported as mean ± standard deviation for continuous variables or percentages of individuals for categorical variables. Normality of data distribution was assessed using the Shapiro–Wilk test. Group comparisons were performed using the two-sample *t*-test and chi-square test for continuous and categorical variables, respectively. One-way analysis of variance was used to compare the three groups (Groups A, B, and C). When a significant difference was found, *post hoc* testing with Bonferroni comparisons was used to identify specific group differences. A *post hoc* power analysis was conducted to estimate the statistical power achieved with the final sample size. Based on the observed effect size for the main finding of the study [difference in GLS between women who experienced LV systolic recovery (Group B) and controls (Group C)], a significance level of *α* = 0.05, and the sample size, the achieved power (1−*β*) was calculated. The analysis indicated a statistical power of 99.6%, suggesting that the study was adequately powered to detect the observed effect.

Reproducibility analyses were previously published by our group (12). To assess intraobserver reproducibility, GLS and MW measurements were repeated in 13 randomly selected individuals by the same operator, who was blinded to the previous measurements. Reproducibility was assessed using the intraclass correlation coefficient (ICC) based on a two-way mixed-effects model for single measurements and absolute agreement. ICC values between 0.75 and 0.90 were considered indicative of good agreement, whereas values >0.90 indicated excellent agreement. Statistical analyses were performed using IBM-SPSS, version 23 (SPSS Inc, Chicago, IL, USA). A *p*-value <0.05 was considered significant.

## 3 Results

### 3.1 Baseline characteristics

**Figure 1:**
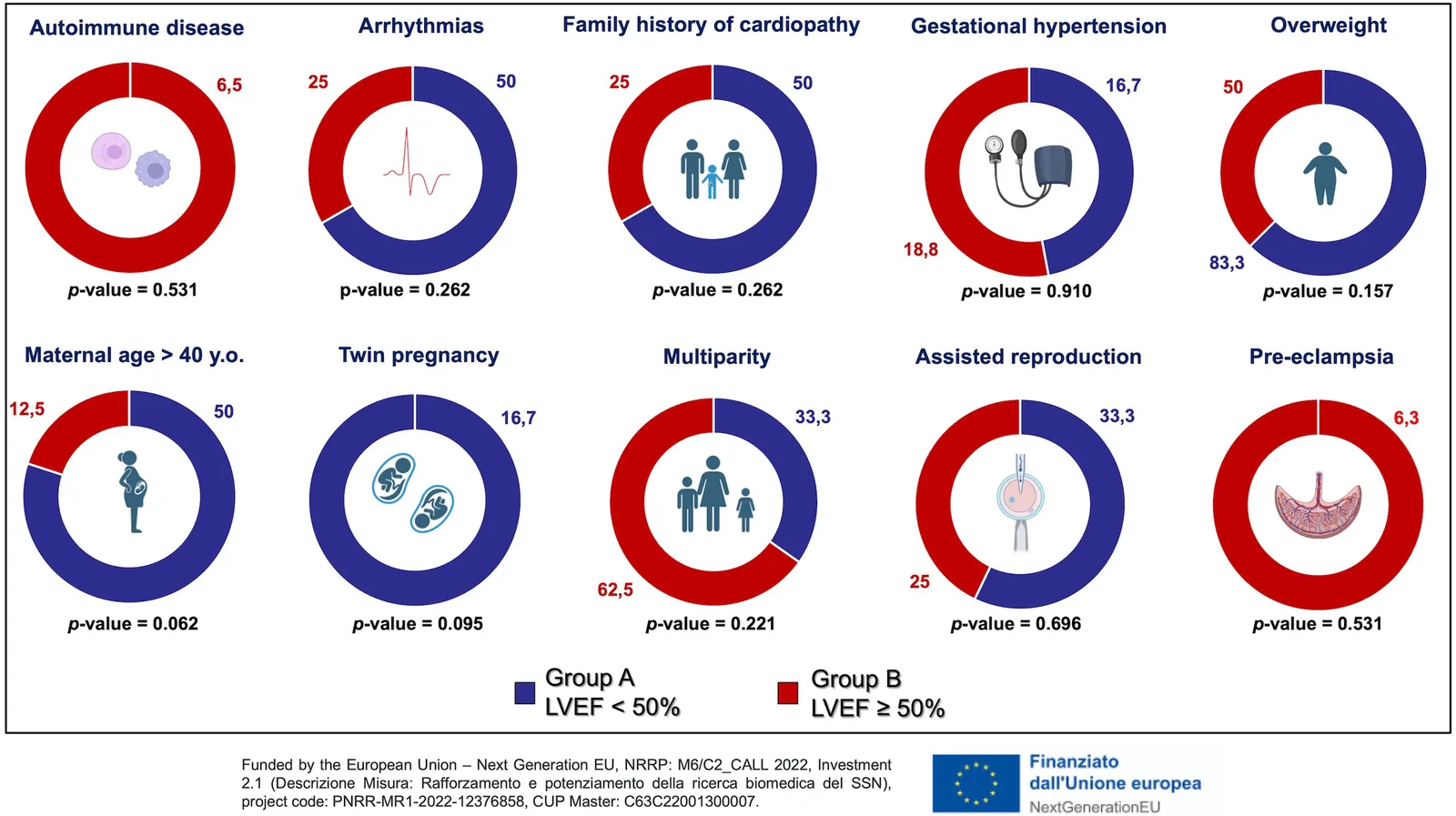
A schematic representation of risk factors associated with peripartum cardiomyopathy in the comparison of Group A (LVEF <50%) and Group B (LVEF ≥ 50%).

Twenty-two patients with a diagnosis of PPCM were analysed in this cohort (mean age 42.1 ± 8.9 years) and compared with 21 healthy women matched for age (Supplementary Figure 1S). Baseline demographic and clinical characteristics of the study cohort, according to LV systolic recovery status, are presented in Table 1. Among patients with PPCM, persistent LV systolic dysfunction was observed in six patients (Group A, 27.2%), whereas 16 (72.7%) showed LVEF recovery (Group B). PPCM presented at the end of pregnancy or during the early postpartum period in 59% of women. Dyspnoea and peripheral oedema were reported in 63.6% and 31.8% of patients, respectively. Hypertensive disorders complicated approximately 18.2% of cases, with pre-eclampsia reported in only two cases. No significant differences in clinical characteristics or PPCM-related risk factors were observed between Groups A and B (Figure 1). A higher, although not statistically significant, prevalence of twin pregnancy (16.7% vs. 0%, *p* = 0.095), late pregnancy presentation (50% vs. 12.5%, *p* = 0.062), and severe obesity (50% vs. 12.5%, *p* = 0.062) was observed in women with persistent LV dysfunction (Group A) compared with those who experienced LVEF improvement (Group B). A more critical onset of PPCM, characterised by congestive HF, was also reported more frequently in Group A than in Group B (50% vs. 12.5%, *p* = 0.062 respectively). Standard HF drugs were administered in both study groups, although diuretics were more commonly used in Group A (83.3% vs. 18.8%, *p* = 0.005, Table 2). Oral anticoagulation was also more frequently prescribed in Group A than in Group B (33.3% vs. 0%, *p* = 0.015, Table 2). None of the patients included in the present cohort received bromocriptine therapy during the acute phase of the disease **(**Table 2).

**Table 1:** Clinical and demographic characteristics of PPCM patients and controls.

Variable	Group A LVEF <50% (*n* = 6)	Group B LVEF ≥50% (*n* = 16)	Group C controls (*n* = 21)	*p*-Value
Age (years)	45.0 ± 10.6	41.1 ± 8.3	40.3 ± 8.0	0.490
Systolic arterial pressure (mmHg)	113.8 ± 15.5	119.7 ± 17.2	116.3 ± 12.5	0.662
Diastolic arterial pressure (mmHg)	71.7 ± 15.1	71.9 ± 9.6	71.8 ± 10.2	0.999
Hypertension [*n* (%)]	4 (66.7)	5 (31.3)	-	0.132
Diabetes mellitus [*n* (%)]	0	0	-	-
Hypercholesterolemia [*n* (%)]	3 (50.0)	2 (12.5)	-	0.062
Congestive heart failure [*n* (%)]	3 (50.0)	2 (12.5)	-	0.062
Endocrine disease [*n* (%)]	1 (16.7)	5 (31.3)	-	0.494
Chronic kidney disease [*n* (%)]	1 (16.7)	0	-	0.105
Tobacco smoking [*n* (%)]	2 (33.3)	3 (18.8)	-	0.467
Time from diagnosis to follow-up (months)	69.7 ± 95.1	74.7 ± 74.8	-	0.897
Number of pregnancies [*n* (%)]				0.067
0	0	0	7 (33.3)	
1	4 (66.7)	13 (81.3)	8 (38.1)	
2	2 (33.3)	2 (12.5)	5 (23.8)	
3	0	1 (6.3)	1 (4.8)	

Values are mean ± SD or *n* (%). LVEF, left ventricular ejection fraction; PPCM, peripartum cardiomyopathy.

**Table 2:** Medications of patients with PPCM and controls at the time of enrolment.

Medication	Group A LVEF <50% (*n* = 6)	Group B LVEF ≥ 50% (*n* = 16)	Group C controls (*n* = 21)	*p*-Value
ACE inhibitors [*n* (%)]	1 (16.7)	6 (37.5)	-	0.350
ARBs [*n* (%)]	1 (16.7)	1 (6.3)	-	0.449
ARNI [*n* (%)]	3 (50.0)	5 (31.3)	-	0.416
Beta-blockers [*n* (%)]	6 (100)	14 (87.5)	-	0.364
CCBs [*n* (%)]	0	0	-	-
MRAs [*n* (%)]	2 (33.3)	4 (25.0)	-	0.696
Diuretics [*n* (%)]	5 (83.3)	3 (18.8)	-	0.005
Antiarrhythmics [*n* (%)]	1 (16.7)	1 (6.3)	-	0.449
Aspirin [*n* (%)]	1 (16.7)	0	-	0.095
Anticoagulants [*n* (%)]	2 (33.3)	0	-	0.015
SGLT2 inhibitors [*n* (%)]	4 (66.7)	7 (43.8)	-	0.788
Bromocriptine [*n* (%)]	0	0	-	-

Values are *n* (%). ACE, angiotensin-converting enzyme; ARB, angiotensin receptor blockers; ARNI, angiotensin receptor-neprilysin inhibitors; CCB, calcium channel blockers; LVEF, left ventricular ejection fraction; MRA, mineralocorticoid receptor antagonists; PPCM, peripartum cardiomyopathy; SGLT2, sodium-glucose cotransporter 2.

### 3.2 Echocardiographic findings

As shown in Table 3, PPCM patients with persistent LVEF < 50% exhibited larger LV volumes and LV end-diastolic diameters compared with patients with improved LVEF (*p* < 0.05 for all) and controls (*p* < 0.001 for all). Left atrial volumes and estimated LV filling pressures were higher in Group A than in the other groups, while PALS was significantly reduced (*p* < 0.001). RV longitudinal systolic function, assessed using tricuspid annular plane systolic excursion (TAPSE) and RV GLS, was also more impaired in Group A than in Groups B and C. When comparing women who had recovered LVEF (Group B) with controls (Group C), LV volumes (*p* < 0.05), end-diastolic diameters (*p* < 0.001), and LV mass (*p* = 0.001) remained significantly increased in Group B, along with reduced relative wall thickness (*p* = 0.012); in addition, a more impaired PALS was detected compared with Group C (*p* < 0.001), associated with a trend towards increased LA volume (*p* = 0.052). A significant reduction of RV GLS was still observed in Group B compared with Group C (*p* = 0.016).

**Table 3:** Echocardiographic variables of Group A, Group B, and Group C.

Variable	Group A LVEF <50% (*n* = 6)	*p*-Value (vs. Group C)	Group B LVEF ≥ 50% (*n* = 16)	*p-*Value (vs. Group C)	Group C controls (*n* = 21)	*p*-Value (between Groups)
LV IVS (mm)	8.3 ± 1.0	0.127	7.7 ± 1.1	0.444	7.4 ± 1.4	0.243
LV PW (mm)	8.2 ± 0.7	0.499	7.3 ± 0.9	0.310	7.7 ± 1.5	0.296
LVEDD (mm)	58.0 ± 8.1	<0.001	50.3 ± 5.3	<0.001	43.8 ± 3.4	<0.001
RWT	0.28 ± 0.05	0.033	0.30 ± 0.04	0.012	0.35 ± 0.07	0.009
LV mass/BSA (g/m^2^)	92.7 ± 32.9	<0.001	73.1 ± 18.0	0.001	55.5 ± 13.1	<0.001
LVEDV (ml)	178.3 ± 80.0	<0.001	109.2 ± 31.1	0.021	90.3 ± 16.0	<0.001
LVESV (ml)	121.3 ± 73.9	<0.001	52.4 ± 17.4	<0.001	33.7 ± 6.9	<0.001
LVEF (%)	34.7 ± 10.3	<0.001	53.0 ± 3.6	<0.001	62.3 ± 4.4	<0.001
LVEF nadir (%)	26.5 ± 12.0	-	33.7 ± 11.8	-	-	0.220
GLS (%)	12.3 ± 5.0	<0.001	18.0 ± 3.1	<0.001	21.8 ± 1.7	<0.001
GWI (mmHg%)	1,081 ± 636	<0.001	1,712 ± 476	0.068	1,954 ± 305	<0.001
GCW (mmHg%)	1,441 ± 713	<0.001	2,058 ± 479	0.061	2,312 ± 319	0.001
GWW (mmHg%)	213 ± 80	<0.001	146 ± 148	0.058	79 ± 45	0.013
GWE (%)	85 ± 7	<0.001	93 ± 6	0.023	96 ± 2	<0.001
PALS (%)	23.7 ± 12.8	<0.001	35.7 ± 7.6	<0.001	46.4 ± 8.0	<0.001
LAVI (mL/m^2^)	40.3 ± 21.3	0.014	31.2 ± 7.6	0.052	26.3 ± 7.2	0.016
E/A	1.9 ± 0.9	0.144	1.4 ± 0.6	0.430	1.5 ± 0.4	0.161
E/e’	12.7 ± 4.7	<0.001	6.9 ± 1.7	0.441	6.5 ± 1.4	<0.001
RV GLS (%)	14.1 ± 4.9	<0.001	18.8 ± 3.8	0.016	21.8 ± 3.0	<0.001
RV FWS (%)	19.7 ± 7.3	0.156	22.0 ± 5.2	0.306	24.1 ± 4.9	0.296
TAPSE (mm)	19.2 ± 5.8	0.008	23.4 ± 2.3	0.159	24.9 ± 3.8	0.007
PAPs (mmHg)	30.0 ± 5.0	0.246	25.1 ± 4.4	0.916	25.3 ± 8.1	0.315

BSA, body surface area; FWS, free wall strain; GCW, global constructive work; GLS, global longitudinal strain; GWE, global work efficiency; GWI, global work index; GWW, global wasted work; IVS, interventricular septum; LAVI, left atrial volume indexed; LV, left ventricle; LVEDD, left ventricular end-diastolic diameter; LVEDV, left ventricle end-diastolic volume; LVEF, left ventricular ejection fraction; LVESV, left ventricle end-systolic volume; PALS, peak atrial longitudinal strain; PAPs, pulmonary artery systolic pressure; PW, posterior wall; RV, right ventricle; RWT, relative wall thickness; TAPSE, tricuspid annular plane systolic excursion. Values are mean ± SD.

**Figure 2:**
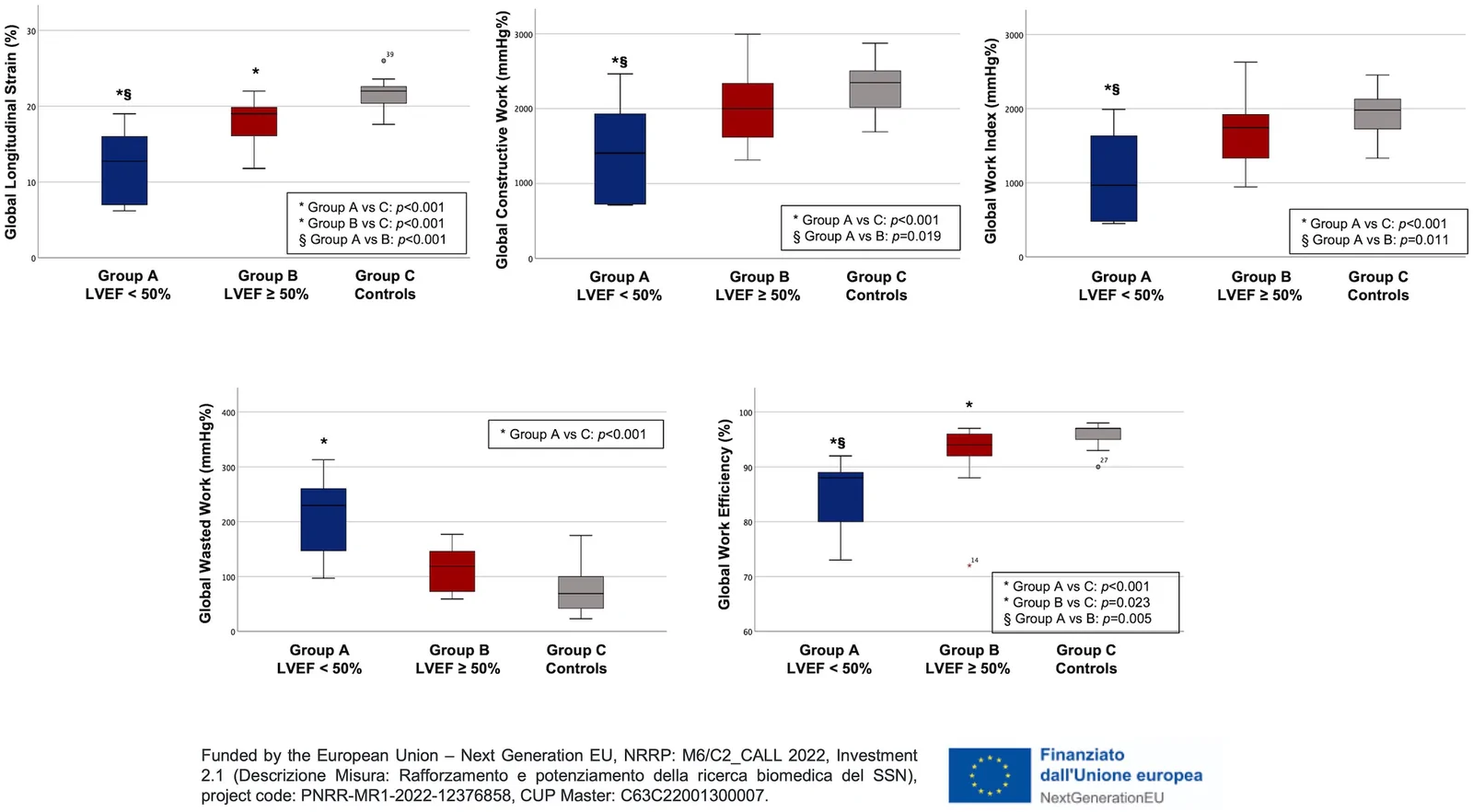
Box plots showing left ventricular (LV) global longitudinal strain (GLS) and myocardial work parameters in women with peripartum cardiomyopathy stratified according to follow-up LV ejection fraction (Group A: LVEF <50%; Group B: LVEF ≥ 50%) and in healthy controls (group C). The panels display GLS, global work index (GWI), global constructive work (GCW), global wasted work (GWW), and global work efficiency (GWE). Patients with persistent LV systolic dysfunction (Group A) exhibited significantly impaired GLS and myocardial work parameters compared with both Group B and controls, with lower GWI and GCW, higher GWW, and reduced GWE. In women who experienced LV systolic recovery (Group B), a significant reduction in GLS and GWE compared with controls (Group C) persisted. Statistical comparisons are indicated in the figure (*Group A and B vs. Group C; §Group A vs. B).

**Figure 3:**
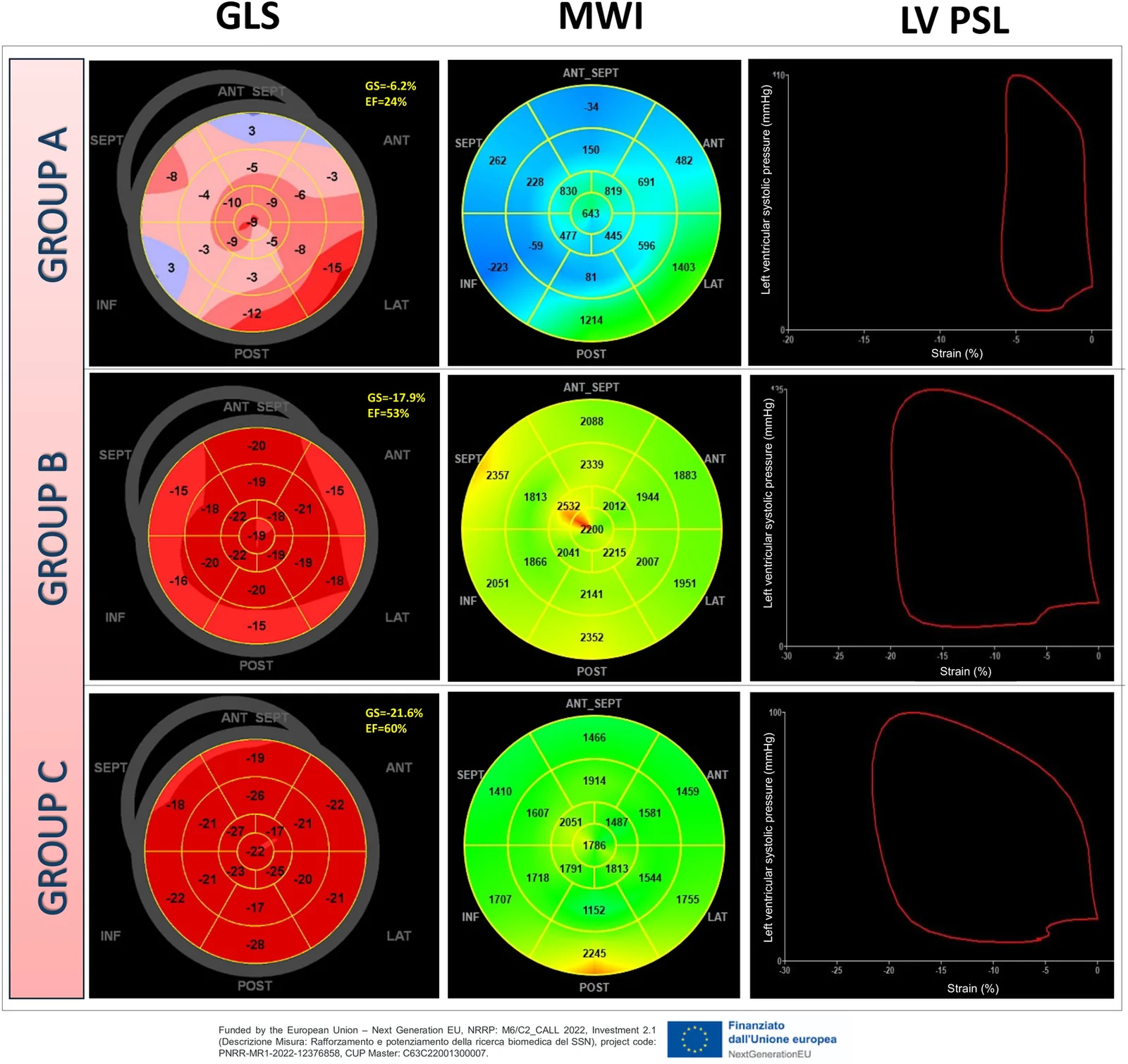
Representative 17-segment bull's-eye representation of global longitudinal strain (GLS, left), myocardial work index (MWI, middle), and global left ventricular (LV) pressure–strain loop (LV PSL, right) in a patient with persistently reduced LVEF (Group A), a patient who experienced recovered LVEF (Group B), and a healthy subject (Group C).

Speckle-tracking analysis showed that GLS, GWI, and GCW were significantly lower in Group A than in Group B and Group C, consistent with the observed LVEF impairment, together with increased GWW and consequent reduction in GWE (Figure 2 and Table 3). Notably, in women who experienced LV systolic recovery (Group B), a significant reduction of GLS (18.0 ± 3.1% vs. 21.8 ± 1.7%, *p* < 0.001) and GWE (92.6 ± 6.0 vs. 95.9 ± 2.0%, *p* = 0.023), compared with controls (Group C), was observed (Figure 2). GWI and GCW also appeared lower in Group B than in Group C, although these differences did not reach statistical significance (GWI: 1,712 ± 476 vs. 1,954 ± 305 mmHg%, *p* = 0.068; GCW: 2,058 ± 479 vs. 2,312 ± 319 mmHg%, *p* = 0.061), with a trend towards higher GWW (146 ± 148 vs. 79 ± 45 mmHg%, *p* = 0.058) in Group B (Figure 3). Given the small sample size, we conducted a *post hoc* power analysis to assess the likelihood of detecting a meaningful effect for one of the study's main findings. Based on the observed difference in GLS between women who experienced LV systolic recovery (Group B) and controls (Group C) (3.8%), the study achieved >90% power at a two-sided *α* level of 0.05. The ICC for intraobserver variability of MW indices was 0.97 for GWI and GCW, 0.89 for GWW, 0.96 for GWE, and 0.98 for GLS (all *p* < 0.001), demonstrating excellent agreement (Supplementary Table 1S).

## 4 Discussion

**Figure 4:**
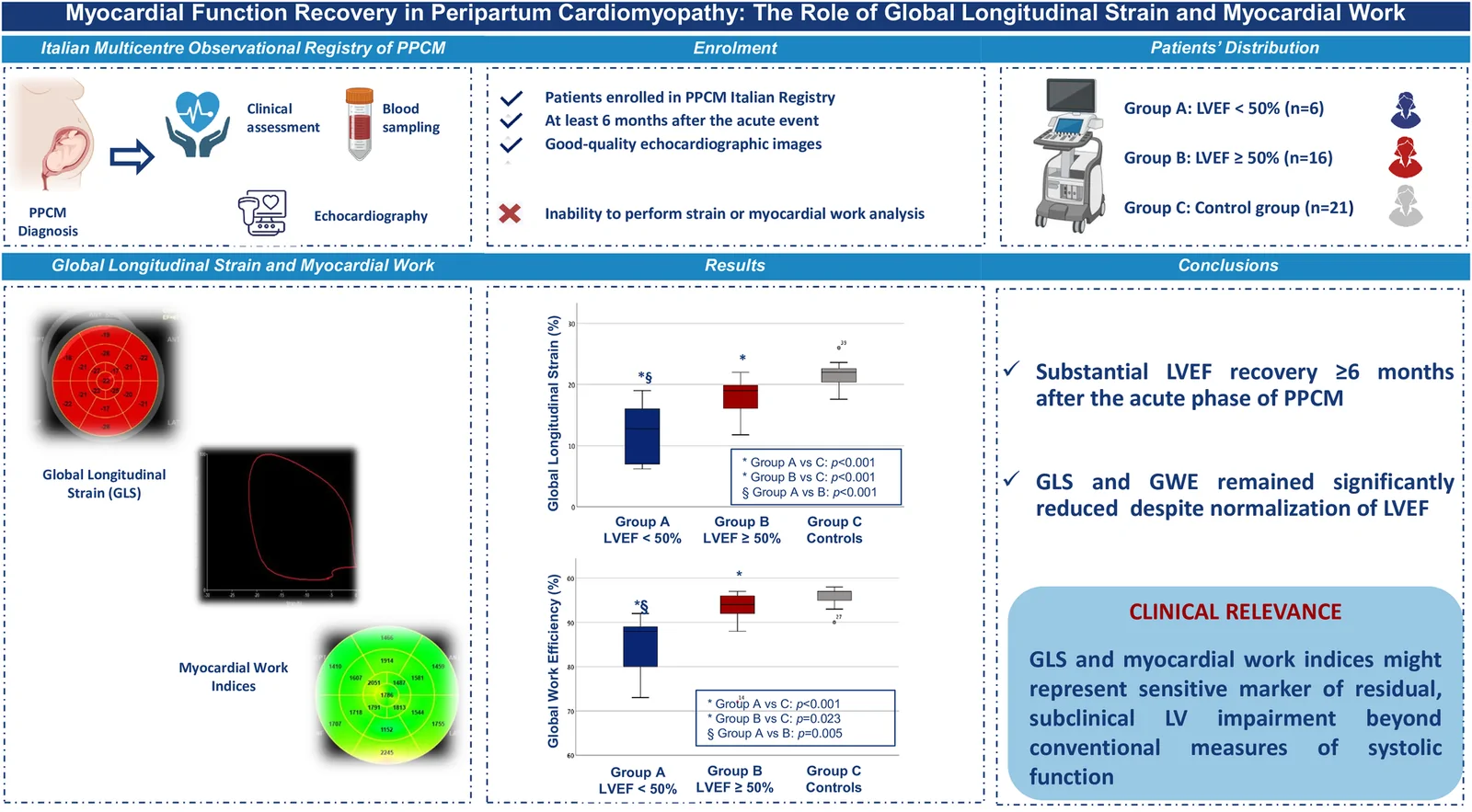
Graphical summary of the study design and main findings. Women with peripartum cardiomyopathy enrolled in the Italian Multicentre Observational Registry underwent clinical and echocardiographic assessment, including global longitudinal strain (GLS) and non-invasive myocardial work analysis, at least 6 months after the acute event. Patients were stratified according to follow-up LVEF into Group A (LVEF < 50%) and Group B (LVEF ≥ 50%) and compared with healthy controls (Group C). Despite LVEF recovery, women in Group B showed persistently reduced GLS and global work efficiency compared with controls.

The present study demonstrates that LVEF recovery occurred in a substantial proportion of women with PPCM after a minimum follow-up of 6 months from the acute phase. Importantly, patients who achieved recovery of LVEF continued to exhibit persistent structural and functional abnormalities compared with healthy controls, including larger LV dimensions and impaired longitudinal function of both the left atrium and the right ventricle. Despite normalisation of LVEF, GLS and GWE remained significantly reduced (Figure 4). The persistence of abnormal GLS and GWE in the presence of recovered LVEF suggests that apparent systolic recovery, as defined by conventional measures, may not reflect complete restoration of LV systolic function. Rather, these findings indicate the presence of residual, subclinical LV dysfunction not captured by LVEF alone. This observation underscores the potential value of myocardial deformation and MW indices as more sensitive markers of persistent abnormalities despite LVEF recovery, with potentially relevant clinical implications for more refined risk stratification, closer follow-up, and prolonged HF therapy. In addition, residual abnormalities in myocardial deformation or MW parameters may have implications for counselling regarding subsequent pregnancies, suggesting that apparent recovery based on LVEF alone may not fully reflect myocardial functional normalisation.

PPCM is increasingly recognised as a complex clinical entity with multifactorial pathophysiology involving genetic susceptibility, inflammation, autoimmune mechanisms, and maladaptive responses to haemodynamic stress and oxidative imbalance (15). Oxidative stress may promote prolactin cleavage into an antiangiogenic fragment that contributes to endothelial dysfunction and microvascular injury (16). These processes may ultimately lead to myocardial deformation abnormalities that are not fully captured by LVEF alone but can be revealed by strain parameters. LVEF has been traditionally considered the most representative parameter of LV systolic function, and, in pregnant women with HF symptoms, a cut-point of <45% has been used as a diagnostic criterion for PPCM. It has been largely demonstrated, in several clinical conditions, that GLS is more sensitive than LVEF in detecting subclinical LV dysfunction (17, 18). In a case series of six women with PPCM, Tamrat et al. showed that GLS impairment may be present prior to HF symptoms and changes in LVEF and LV dimensions (4). This would suggest a potential role for GLS during pregnancy in identifying women at increased risk of developing PPCM. Furthermore, the prognostic role of GLS and global circumferential strain in women with PPCM has also been recognised, with a more impaired LV global strain at presentation associated with a higher incidence of lack of LV recovery, death, or LV assist device implantation (19).

In this study, we expand the current knowledge on PPCM by providing further insights into the myocardial performance of women who experienced PPCM, using advanced echocardiographic parameters such as GLS and MW indices. As expected, in the group of patients with persistent systolic dysfunction (Group A), a more dilated LV was associated with a more impaired GLS, reflecting higher wall stress and impaired myocardial relaxation. LV remodelling was also associated with altered energy metabolism, detectable by non-invasive MW analysis, revealing significantly impaired GWI and GCW, increased wasted work, and a consequent significant reduction of GWE. These findings seem to be even more meaningful than GLS reduction in patients with PPCM, considering that non-invasive MW estimation—through the integration of myocardial deformation and afterload—has demonstrated incremental value in evaluating myocardial function, especially under conditions of increased afterload. LA volume was significantly increased in Group A, accompanied by a marked reduction in PALS, which strongly represented a marker of higher filling pressure and diastolic dysfunction, supported by significantly increased E/e' values. Alongside LV dysfunction, the right heart chambers showed a significantly reduced longitudinal systolic function, detected by an impairment of TAPSE and RV GLS, while RV free wall strain (FWS) remained relatively preserved. This apparent discrepancy may be explained by the fact that these parameters reflect different aspects of RV mechanics. TAPSE represents a one-dimensional measure of tricuspid annular displacement and is strongly influenced by ventricular geometry and loading conditions. The reduction in RV-GLS may partly reflect the coexisting LV and interventricular septal dysfunction, because septal segments contribute to the calculation of global RV longitudinal strain, whereas RV FWS may remain preserved because of compensatory mechanisms, providing a more direct assessment of myocardial deformation.

Consistent with findings from the European Society of Cardiology EURObservational Research Programme PPCM registry, after the acute phase of disease, we observed LV systolic recovery in approximately 70% of patients (20). However, despite normalisation of LVEF, GLS remained impaired, suggesting that conventional measures of systolic function may underestimate persistent myocardial injury. The persistence of abnormal GLS may reflect residual structural alterations related to prior inflammation, myocardial oedema, or fibrotic remodelling. In this subgroup of patients with recovered systolic function (Group B), the persistent reduction in PALS compared with healthy controls is likely more closely related to impaired myocardial deformation than to atrial chamber enlargement, further supporting the close relationship between PALS and GLS and confirming the role of PALS as a marker of subclinical LV dysfunction (21).

In line with our findings, previous studies have reported impaired global and segmental longitudinal strain in PPCM patients with preserved LVEF, with strain values intermediate between those observed in patients with overt PPCM and healthy controls. Although GLS did not predict HF hospitalisation in that small cohort, it highlighted the presence of subclinical systolic dysfunction despite apparent recovery (2). Importantly, our study extends these observations by demonstrating, for the first time, that GWE also remains significantly reduced in PPCM women with recovered LVEF compared with controls. As GWE integrates myocardial deformation with afterload, its impairment suggests persistent abnormalities not only in myocardial mechanics but also in myocardial energetic performance. Together, these findings support the concept that myocardial recovery in PPCM may be incomplete at both functional and metabolic levels, even when LVEF has normalised.

In a recent study, Meledin et al. reported reduced GLS and MW indices across the entire PPCM cohort, without significant differences between patients with recovered and non-recovered LVEF (19). The lack of discrimination between groups was hypothesised to reflect a potential underestimation of MW in dilated ventricles, where increased wall stress may affect the accuracy of non-invasive pressure–strain-derived parameters. In our study, patients with PPCM likewise exhibited larger LV dimensions compared with controls. Nevertheless, we observed a clear trend towards progressive improvement in myocardial performance from Group A to Group C, suggesting partial functional recovery along the clinical spectrum. Although the difference between Groups B and C did not reach statistical significance, this finding may be partly explained by limited statistical power because of the relatively small sample size. Overall, our results support the concept that deformation and MW parameters may capture gradations of recovery, even in the presence of persistent ventricular dilation. Indeed, while residual chamber enlargement provides information on left ventricular remodelling, it does not necessarily reflect intrinsic myocardial performance, which can be studied by advanced echocardiographic parameters. Importantly, the ability of MW indices to characterise myocardial mechanics despite ventricular dilatation is supported by studies in patients with dilated cardiomyopathy (22, 23). In these populations, GCW and GWE have been shown to improve following optimised heart failure therapy and to independently predict major adverse cardiovascular events (22). Therefore, despite the potential influence of chamber geometry on pressure–strain-derived indices, our findings reinforce the ability of MW analysis to provide clinically meaningful information even in the setting of ventricular remodelling.

Although abnormalities in myocardial strain have long been recognised in PPCM, the persistence of reduced GLS and impaired GWE despite normalisation of LVEF warrants further investigation to better elucidate the underlying pathophysiological mechanisms and their clinical implications. A potential hypothesis is the presence of diffuse, microscopic fibrosis resulting from prior myocardial inflammation, oedema, or injury during the acute phase. Persistent impairment in myocardial deformation may be driven by more subtle structural alterations or by functional abnormalities involving myocardial energetics, cytoskeletal integrity, or microvascular dysfunction (24). However, these mechanisms remain speculative at present, as no cardiac magnetic resonance data were available in our cohort. Integration with cardiac magnetic resonance imaging would be valuable to investigate whether abnormalities in myocardial deformation are associated with the presence of myocardial scarring, interstitial, or replacement fibrosis. Furthermore, the incorporation of metabolomic and proteomic profiling may provide additional insights into the underlying inflammatory burden and help to better characterise the biological mechanisms responsible for persistent myocardial abnormalities in women with PPCM.

## 5 Study limitations

This study has some limitations that should be acknowledged. The relatively small sample size, inherent to the rarity of PPCM and the single-registry cohort, limited statistical power and may have precluded the detection of differences in selected MW parameters between groups. However, we performed a power calculation using GLS difference between Group B and Group C, and, despite the small sample size, the large effect size yielded very high power (Supplementary Figure 2S). The retrospective design and cross-sectional echocardiographic assessment performed at follow-up prevented evaluation of longitudinal changes in myocardial deformation and work indices. Moreover, because baseline strain and MW measurements during the acute PPCM phase were unavailable for all patients, our findings only demonstrate persistent abnormalities at follow-up and do not allow formal assessment of myocardial recovery trajectories. NT-proBNP values were not systematically available in all patients, precluding assessment of the association between residual abnormalities in myocardial deformation and MW parameters and circulating biomarkers. Exclusion of patients with unavailable or suboptimal image quality for deformation analysis may have introduced a selection bias and may have limited the generalisability of our findings. Another limitation is the wide range of follow-up duration (6–240 months, mean time 73.3 ± 78.5 months), reflecting the design of the Italian multicentre PPCM registry, which included both newly diagnosed patients and women with a remote history of the disease. Although follow-up duration did not significantly differ between groups, it represents a potential source of heterogeneity and may have influenced the observed echocardiographic findings. Finally, clinical outcome data were not analysed, preventing assessment of the prognostic implications of persistent GLS and GWE abnormalities. Larger prospective studies with longitudinal multimodality imaging and outcome evaluation are warranted to confirm these findings.

## 6 Conclusions

GLS and MW indices provide a more comprehensive assessment of myocardial performance in patients with PPCM, irrespective of LV systolic recovery as defined by LVEF. The persistence of impaired GLS and GWE in women with normalised LVEF suggests that these parameters may serve as sensitive markers of residual LV dysfunction not captured by conventional measures. Identification of this subgroup may have important clinical implications, potentially supporting closer surveillance, prolonged HF therapy, and more tailored management strategies. Furthermore, these findings suggest that recovery of LVEF alone may not be sufficient to define true myocardial recovery in PPCM and may have relevant implications for risk stratification in subsequent pregnancies. Larger, prospective studies are warranted to further elucidate the underlying pathophysiological mechanisms of persistent dysfunction in PPCM and to determine the prognostic value of deformation and MW indices in this clinical setting.

## Data Availability

The raw data supporting the conclusions of this article will be made available by the authors, without undue reservation.
