# Supplementary material for: Myocardial function recovery in peripartum cardiomyopathy: the role of global longitudinal strain and myocardial work
Source: Front Cardiovasc Med. 2026 Aug 26;13:1860070. doi: 10.3389/fcvm.2026.1860070 (PMC13553815; doi:10.3389/fcvm.2026.1860070)
Supplement: Supplementary file 1 [file Table1.docx]

SUPPLEMENTARY MATERIAL

**Supplementary Table 1S: Intra-observer reproducibility of LV global longitudinal strain and myocardial work indices assessed by interclass correlation coefficient.**

| Variable | Interclass Correlation Coefficient  (95% CI) | *P* value |
| --- | --- | --- |
| Global longitudinal strain (%) | 0.98 (0.93-0.99) | <0.001 |
| Global work index (mmHg%) | 0.97 (0.90-0.99) | <0.001 |
| Global constructive work (mmHg%) | 0.97 (0.91-0.99) | <0.001 |
| Global wasted work (mmHg%) | 0.89 (0.69-0.97) | <0.001 |
| Global work efficiency (%) | 0.96 (0.88-0.99) | <0.001 |
